# Research for improvement of care for pediatric heart transplant patients

**DOI:** 10.1016/j.jhlto.2025.100397

**Published:** 2025-09-24

**Authors:** Alicia Kamsheh, Janet Scheel

**Affiliations:** Division of Pediatric Cardiology, Washington University School of Medicine, St. Louis, MO

**Keywords:** Pediatric heart transplant, Pediatric research, Pediatric research regulations

## Abstract

In the past nearly-six decades since the first pediatric heart transplant, the field has made great strides. Yet, there remains substantial room for improvement. Wait time, graft longevity, cardiac allograft vasculopathy, rejection monitoring, and balancing immunosuppression with side effects remain significant challenges and affect patient quality of life. We review milestones in pediatric heart transplant research, including the formation of transplant registries, major multicenter prospective studies, and the first randomized clinical trial in pediatric heart transplantation. We discuss ongoing challenges to research in this field, including small population size, heterogeneity, and difficulty with identifying appropriate endpoints. We examine historical regulatory barriers and how they have been addressed to date. Future directions, including key areas of focus and funding pathways are also discussed.

Since the first reported infant heart transplant in 1967, there have been considerable strides made in the field of pediatric heart transplantation.^1^ While this initial infant recipient survived less than a day after transplant, today’s pediatric heart transplant recipients have a 1-year survival and 5-year survival conditional on surviving to 1-year greater than 90%.^2^ Registry data from the International Society for Heart and Lung Transplantation (ISHLT), demonstrates incremental improvement in the freedom from death or retransplant for pediatric heart recipients during the last three transplant eras (ie, 1992-2000, 2001-2009, 2010-2017).^3^, ^4^ However, despite this progress, there remains significant room for improvement in the care and outcomes of these patients, and major advances in pediatric heart transplant research that improves the quality of life for our patients are sparse.

Numerous challenges continue to persist in pediatric heart transplantation. First, wait times are long, often necessitating long hospital stays. Second, rejection remains an ongoing concern and, despite some advances, the gold standard for testing for rejection is still invasive. The side effects of immunosuppression are also a major issue, which leads to accumulating morbidity in young patients over time. Further, our ability to diagnose, treat, and prevent cardiac allograft vasculopathy (CAV) remains suboptimal. Last, but certainly not least, the lifespan of the transplant graft remains shorter than an average lifespan in a non-transplanted pediatric patient.

## Pediatric heart transplant research to date

With the passing on the National Organ Act (NOTA) of 1984, the Scientific Registry for Transplant Recipients (SRTR) was established.^5^ It is operated under a contract from the United States Department of Health and Human Services and is required to support the ongoing evaluation of the scientific and clinical status of solid organ transplantation. Its data is largely collected from the Organ Procurement and Transplantation Network (OPTN). OPTN collects data from transplant programs, organ procurement organizations, and histocompatibility labs. It is required to make all data available to the public while honoring privacy rules. It was not created as a primary research tool and is limited by the data that it collects. For example, it does not differentiate between specific congenital heart disease lesions or surgical stages.

The ISHLT created a registry for thoracic organ transplant and has provided outcomes data annually.^6^ It is a more granular database collecting data on multiple risk factors in addition to outcomes and survival. It has multiple international registry partners. It is challenged by its volunteer nature, making the completeness and accuracy of its data unknown. It is also challenged by international laws regarding data collection and recently had a brief hiatus as it became compliant with the EU Data Governance Act.

At the present time, many of the studies in the field are the result of the Pediatric Heart Transplant Study (PHTS). PHTS was founded in 1993 and renamed the Pediatric Heart Transplant Society (PHTS) in 2018.^7^ The PHTS Registry is an event-driven database containing comprehensive, prospectively collected data from pediatric heart transplant recipients at 64 participating centers. To date, it has enrolled 13,409 patients at listing and will soon contain data from 10,000 pediatric heart transplantations.^8^ Studies utilizing data from this registry have resulted in 131 publications and 170 presentations and abstracts on a broad range of topics, including the effect of induction therapy on rejection and survival, outcomes in patients with Fontan physiology, and the outcomes with a positive retrospective crossmatch.^9^, ^10^, ^11^ The PHTS has been an excellent example of what can be accomplished when centers treating a small population of patients with a rare disease join to conduct research. With the development of a PHTS Data Coordinating Center (KIRSO), it is now possible to link PHTS data with other registries. It can also help with planning for prospective studies, including study design, power calculations and development and validation of surrogate endpoints.^12^ However, there remains inherent limitations to registry-based studies, including the inability to account for the multitude of possible confounders, missing data, and a lack of long-term follow-up.

There have been several other important research studies that have had a major impact on our field. The Clinical Trials in Organ Transplantation in Children project was a cooperative research program sponsored by the National Institute of Allergy and Infectious Diseases.^13^ The purpose was to improve outcomes in children who had undergone solid organ transplants. CTCOTC-04 was a multicenter, prospective observational study to determine the impact of alloantibodies on heart transplant outcomes.^14^ The Berlin Heart EXCOR IDE clinical study was a prospective, multicenter, single-arm clinical cohort study with a propensity-matched historical control group supported with ECMO.^15^ It was the first bridge to transplant ventricular assist device study designed for children and led to approval of the Berlin Heart EXCOR by the U.S. Food and Drug Administration.^16^ Finally, the TEAMMATE trial remains the only multicenter randomized clinical trial for immunosuppression used in pediatric heart transplant patients.^17^ This trial compared the use of Everolimus with low-dose tacrolimus to standard-dose tacrolimus with mycophenolate mofetil in this population.

## Challenges and opportunities in pediatric heart transplant research

With decades of research, we have seen some improvement in early transplant survival, although this appears to be plateauing. Long-term survival remains largely unchanged.^4^ If we are going to move our field forward and improve the lives of our patients, we need to continue to pool our data, increase the number of prospective studies, and perform more randomized controlled trials. So why has it been so hard to conduct significant trials and develop improved therapies in this group of patients? What would make it easier?

Drug companies are often unenthusiastic about the investment in a small high-risk population. Often drugs are used off label or as “one-offs.” This does not encourage clinical trial completion or data collection and analysis in our population. It is expensive to include pediatric centers in existing studies, and our patients are heterogeneous, with outcomes varying by age. Given this challenge, the Food and Drug Administration passed the Pediatric Research Equity Act in 2003.^18^ It has allowed multiple sources of data to be used to support pediatric approvals, including extrapolation of adult data and real-world evidence. This change has been particularly valuable in the development of pediatric devices. The Real-World Evidence program provides a new pathway to approval where data can be collected, stored, and curated. That data can then be used to bypass traditional randomized controlled trials in pediatrics, which are both costly and challenging to execute. Using data that had already been collected in the ACTION registry, the FDA (Food and Drug Administration) approved pediatric labeling for the HeartMate 3.^19^ This success then led to the completion of the Berlin Heart Active Driver Trial utilizing the ACTION Network, which became the first prospective pediatric device trial completed within a registry.^20^

To aid in obtaining pediatric drug labeling, pharmaceutical companies have developed pediatric investigational plans (PIPs). These PIPs are submitted to the European Medicines Agency and are aimed at ensuring that the necessary data is obtained to support the development of medicines for pediatric patients. Since 2020, 1866 of these PIPs is have been put forth, though only 12% have been regarding cardiac medications.^21^ Of the 12%, only 14% resulted in positive decisions. Similarly, the FDA requires submission of a pediatric study plan. There are three stages of FDA approval. The Pilot study must show that it is feasible and works. This is followed by Pivotal trials and finally post-market surveillance to ascertain optimal use and safety risk. The Best Pharmaceuticals for Children Act granted a 6-month extension on exclusivity to encourage more pediatric trials. It is encouraging that both of these agencies recognize the importance of this work in children.

In Europe, efforts are underway to integrate real-world data into the regulatory framework for the development, approval, and lifecycle management of pediatric devices. The European Medicines Regulatory Network is working toward the establishment of a sustainable framework for better integration of this data to evaluate products throughout their lifecycle. In 2022, the Data Analysis and Real-World Interrogation Network (DARWIN EU) was established to provide robust RWD analysis tools.^22^ In 2024, the European Medicines Agency issued new guidance for incorporating real-world data into studies.

### Despite these significant efforts, progress remains slow

One of the biggest challenges is finding a reasonable endpoint in a relatively small population that is high risk but has few adverse events. Researchers have tried to overcome this hurdle by creating novel, composite scores to use as a surrogate endpoint. This was the approach used in the TEAMMATE study with the use of the Major Adverse Transplant Event (MATE) score at 30 months.^17^ This was a score developed and validated using data from the PHTS with six components: cellular rejection (ACR), antibody mediated rejection, infection, coronary artery vasculopathy (CAV), post-transplant lymphoproliferative disease, and chronic kidney disease.^12^ Using this score, safety was established, and using three components of the score (CAV, chronic kidney disease, ACR), non-inferiority could be shown at 30 months. Although an efficacy endpoint was not met, several important secondary endpoints were met, including a decrease in donor-specific antibodies, improved renal function, and decreased CMV.

Another approach that has been used in other studies is the probability of benefit as an endpoint. Such endpoints can be imaging, biomarkers, or quality of life measures. Alternatively, we can extrapolate efficacy from adults to children. This works best when the disease process is similar. However, it is clear that the immune system in infants and children is very different from the mature immune system in adults, and thus ignoring this fact could result in missed opportunities for our patients.

Leveraging new tools such as artificial intelligence and machine learning may help us make our research more impactful. Artificial intelligence can help identify eligible patients for study inclusion, interpret imaging as well as analyze and disseminate results. It may help collect and interpret real world data more efficiently and accurately. AI-driven algorithms process large-scale, heterogeneous data to identify patterns and generate predictive models. It can also simulate trial scenarios. In pediatric cardiology, AI is presently being applied to imaging and surgical outcome prediction models. Research suggests that it may be helpful predicting 1-year mortality in the pediatric heart transplant population.^23^, ^24^ However, it is imperative that this technology is used responsibly, as it has the risk of amplifying biases and generating inaccurate results. Close regulation will be imperative.^15^

## Key areas of focus

To address the long wait times, we need to increase our donor pool and donor utilization. We need to push the boundaries of ABO-incompatible transplantation, including the acceptable age range and level of ISO titers. Future research can perhaps make ABO type irrelevant for heart transplant recipients.

We will need to expand the utilization of donation after cardiac death grafts as well as grafts from high-risk donors, including those with hepatitis C. Our organ discard rate in the United States approaches 50%.^25^ Continued study may help us understand which organs being discarded could be safely transplanted. One strategy for this would be increased use of perfusion tools to identify donor hearts that will recover function. The first step is developing ex vivo perfusion tools that can be used for organs from patients weighing less than 40 kg. Artificial intelligence may also help us better analyze the donor information and potential outcomes.

Xenotransplantation is another potential avenue for increasing organ availability. Dr. Leonard Bailey performed a xenotransplant using a baboon in the 1980s.^26^ The outcome was disappointing. More recently, there have been attempts to use genetically modified pig hearts for transplantation. Both recipients died within a couple of months, but this experience will likely lead to further improvements in the methods used. Recently, Mitchell et al published results of an infant model of cardiac xenotransplantation using pig hearts transplanted into baboons.^27^ The outcomes were limited by inflammation rather than cardiac function. With these studies, new immunology knowledge and techniques, perhaps we will usher in an era of xenotransplant.

Improving mechanical support options may decrease or eliminate the need to undergo heart transplantation. Over the past two decades, the use of mechanical support has become routine. Through the Advanced Cardiac Therapies Improving Outcomes Network (ACTION), we have been able to significantly decrease the stroke rate of our patients on these devices and improve their outcomes.^28^ ACTION is a learning network that successfully brought “everyone” together, including patients and their caregivers, regulatory agencies, providers and institutions, researchers, innovators, and industry representatives. Its approach is both simple and effective. It defines a problem, implements quality improvement, and collects real-world evidence in a registry. As already mentioned, that real-world evidence led to the FDA expansion of the labeling for the HeartMate 3 in pediatrics. This was the first time that registry data had been used to expand labeling of a device to pediatrics.^29^

The adult population started using mechanical circulatory support devices for destination therapy many years before us. It has become clear that these devices can support patients well for many years. As they continue to improve, it is also possible that they will be better than the “bio-ventricular assist device”, a transplanted heart. We may ultimately have an artificial heart that lasts longer than the transplanted human heart. This will eliminate wait time since the devices will be readily available with a potentially unlimited supply.

New tools to diagnose rejection are already being used in our clinical practice. We are becoming increasingly comfortable with the use of cell-free deoxyribonucleic acid and molecular biopsies. Both Richmond and Khush have shown excellent correlation with biopsy proven rejection.^30^, ^31^ A more recent article by Jimenez-Blanco et al suggests cell-free deoxyribonucleic acid is most reliable when combined with other biomarkers.^32^ We are not there yet but are getting closer to non-invasive testing that we can be confident tells us what is happening in our patient’s grafts. Our patients will be thrilled when a simple blood draw is all that is needed to diagnose rejection.

Ultimately, perhaps immunosuppression can be tailored to decrease the risk of rejection with fewer medication side effects, including end-organ toxicity and risk of post-transplant lymphoproliferative disorder and other malignancies. We may be able to use personalized medicine to predict those that are more likely to experience rejection, catch it early, and potentially decrease the late graft dysfunction.

CAV remains the leading cause of graft loss in our patients. A better understanding of the pathophysiology of CAV as well as methods for early detection and treatment will hopefully prolong graft survival. Intravascular ultrasound is widely used in the adult transplant patient. It is more challenging in our smaller grafts and has not been widely accepted. In adults, cardiac hybrid positron emission tomography/computed tomography has been used to screen for CAV.^33^ More recently, the addition of visually estimated coronary artery calcium appears to be an independent risk factor for death or retransplant when added to positron emission tomography/computed tomography.^34^ It remains to be seen which of these methods will prove beneficial in detecting clinically significant pediatric CAV.

## Funding the path forward

Just as we need to be thoughtful in the way we design studies and which questions we ask, we will need to continue to look for novel ways to fund our studies. As the future of federal funding becomes uncertain, we will need to look to nonprofits such as Additional Ventures and Enduring Hearts (EH). The Pediatric Heart Transplant Alliance, a collaboration among leading organizations in pediatric heart transplantation, was announced in April 2025. The Alliance brings together six organizations. Founding partners include EH, the ISHLT, PHTS, Transplant Families, ACTION, and Additional Ventures. The mission of the Alliance is to serve as a powerful coalition spotlighting the need for advancements in pediatric heart transplantation through research, education, and advocacy. It also aims to expand access to care, optimize donor organ utilization, and improve post-transplant quality of life. There have already been collaborative grants from EH with ISHLT, EH with PHTS as well as EH with the American Heart Association.^35^ It is in working together in this way that we can successfully move our field forward.

The goal of our field should be to get to a point where money is allocated for pediatric studies, devices and drugs are routinely tested and labeled in pediatrics, high-quality data is collected efficiently, and regulatory issues are streamlined. This will take hard work, collaborative and thoughtful research design, thinking outside the box, and ongoing advocacy for research in our population.

“The best way to predict the future is to create it.” Abraham Lincoln.

## Declaration of Competing Interest

The authors declare that they have no known competing financial interests or personal relationships that could have appeared to influence the work reported in this paper.
